# Interchain Hydrodynamic Interaction and Internal Friction of Polyelectrolytes

**DOI:** 10.1021/acsmacrolett.3c00409

**Published:** 2023-08-25

**Authors:** Ekaterina Buvalaia, Margarita Kruteva, Ingo Hoffmann, Aurel Radulescu, Stephan Förster, Ralf Biehl

**Affiliations:** † Jülich Centre for Neutron Science JCNS and Institute of Biological Information Processing IBI, Forschungszentrum Jülich GmbH, 52425 Jülich, Germany; ‡ Institut Max von Laue-Paul Langevin (ILL), 71 Avenue des Martyrs, CS 20156, F-38042 CEDEX 9 Grenoble, France; § Jülich Centre for Neutron Science JCNS at Heinz Maier-Leibnitz Zentrum (MLZ), Forschungszentrum Jülich GmbH, 85748 Garching, Germany

## Abstract

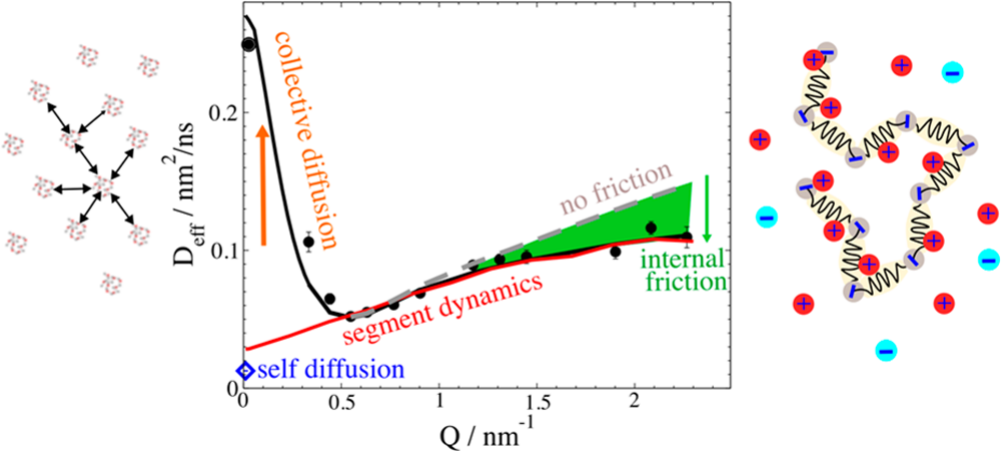

Polyelectrolytes (PE) are polymeric macromolecules in aqueous solutions characterized by their chain topology and intrinsic charge in a neutralizing fluid. Structure and dynamics are related to several characteristic screening length scales determined by electrostatic, excluded volume, and hydrodynamic interactions. We examine PE dynamics in dilute to semidilute conditions using dynamic light scattering, neutron spinecho spectroscopy, and pulse field gradient NMR spectroscopy. We connect macroscopic diffusion to segmental chain dynamics, revealing a decoupling of local chain dynamics from interchain interactions. Collective diffusion is described within a colloidal picture, including electrostatic and hydrodynamic interactions. Chain dynamics is characterized by the classical Zimm model of a neutral chain retarded by internal friction. We observe that hydrodynamic interactions are not fully screened between chains and that the internal friction within the chain increases with an increase in ion condensation on the chain.

Polyelectrolytes (PE) are widespread in nature like RNA or intrinsically unfolded proteins and widely used in technical applications.^1^ Beyond polymer architecture and solvent affinity, mainly the intrinsic charge with the surrounding screening aqueous solvent determines the polymer conformation between a collapsed coil conformation for high salt and a rodlike, at least strongly expanded, conformation if the polymer charges are not screened. Additional hydrogen bonding, dipolar interactions, ion condensation, or chain connectivity influence the collective behavior of the polymer chains. The polymer conformation and the resulting interchain interactions, that may also be entangled at higher PE concentrations, affect macroscopic properties as viscosity, turbidity, collective diffusion, or the interaction with surfaces. General relationships between self-and collective diffusion coefficients *D*_s_ and *D*_c_, radius of gyration *R*_g_, viscosity η are described within the double screening model of Muthukumar^2^ or the scaling model of Dobrynin^3^ considering screening of electrostatics, excluded volume and hydrodynamic interactions (HI). The respective scaling relations present different behavior dependent on low or high salt conditions and concentration in dilute, semi dilute and entangled regimes also dependent on assumptions as hydrodynamic screening between chains.^4^ Experimental data show accordance to the scaling relations but also discrepancies are reported.^5−7^ There has been a need to understand the link of segment level dynamics to chain dynamics since the earliest PE studies.

Hayter et al. examined polystyrene sulfonate (PSS) as the classical PE and discussed the correlation *D(Q)= kTμ(Q)/S(Q)* between PE structure factor (SF) *S(Q*), diffusion coefficient *D(Q)* and single chain mobility *μ(Q)* for low salt concentration.^8^ This quite general relation connects the observed collective diffusion with forces *kT/S(Q)* at thermal energy *kT* between particles or chains expressed in *S*(*Q*) and is related to “de Gennes narrowing”, describing the reduced mobility at the correlation maximum. For colloidal spherical particles Ackerson et al. introduced a reduced mobility at increased concentrations.^9^ Later the δγ-expansion of Beenakker and Mazur quantified this and introduced the hydrodynamic function *H*(*Q*) describing the reduced mobility of hard spheres with increasing concentration splitting into a *Q*-dependent distinct part *H*_d_(*Q*) and a self-part as the reduced self-diffusion *D*_s_/*D*_0_.^10,11^ This results in the well-known correction *D*(*Q*) = *D*_0_*H*(*Q*)/*S*(*Q*) with single particle diffusion *D*_0_, which is derived for spheres but works also for globular proteins, highly anisotropic proteins like antibodies or intrinsically disordered proteins (IDP).^12−20^

The dynamics of a neutral chain on a molecular level is theoretically depicted as Rouse like if hydrodynamic screening is assumed, or Zimm like if hydrodynamic interactions are relevant.^2,21,22^ A direct access to the PE dynamics on a molecular level is possible using neutron spinecho spectroscopy (NSE) which is a unique technique for assessing the polymer chain on the nanometer and nanosecond scales. It has been successfully used to examine polymer dynamics,^23^ protein domain motions,^14,24^ IDP^16,25^ but is rarely applied for PE dynamics.^8,26^

In this Letter, we address the relationship between the macroscopic properties, in particular collective diffusion from dynamic light scattering (DLS) and self-diffusion from pulsed field gradient (PFG) NMR, and chain dynamics on a segmental level observed by NSE for a model PE in aqueous solution. The chain dynamics are described by the Zimm model including internal friction (ZIF). The transition to collective diffusion is governed using the δγ-expansion introducing a HI screening radius *R*_HI._ We demonstrate that the dynamics of charged PE can be fully described assuming a decoupling of local chain dynamics from polymer interchain interactions related to HI and charges for dilute to semidilute PE solutions.

We use PSS acid of molecular weight *M*_w_ = 39 kg/mol (39k) and *M*_w_ = 17.5 kg/mol (17.5k) purchased from Polymer Source Inc., Canada, with *M*_w_/*M*_n_ = 1.03, respectively 1.09 and sulfonation degree > 90% after dialysis to remove impurities. PSSNa samples were titrated with NaOD to pD 7 in D_2_O while PSSH samples were dissolved in D_2_O resulting in pD 2.3 for 10 mg/mL PSSH and pD 1.8 for 30 mg/mL. NaCl was added to increase the ion concentration *c*_I_. Using DLS (Zetasizer Nano ZS, Malvern), we determined the collective diffusion coefficient *D*_c_ of PSS.^27,28^ For low *c*_I_ the signal splits into a fast component due to collective diffusion and a slow component attributed to aggregates. In the further discussion, we always refer to the fast component. Figure 1 shows respective *D*_c_ for PSS 39k at different salt concentrations *c*_s_ with *c*_I_ between 10 mM and 2 M together with related self-diffusion coefficients *D*_s_ measured by PFG NMR. For 17.5k, see Supporting Information (SI). *D*_s_ was measured by using a 600 MHz Varian spectrometer equipped with a diffusion probe head.

**Figure 1:**
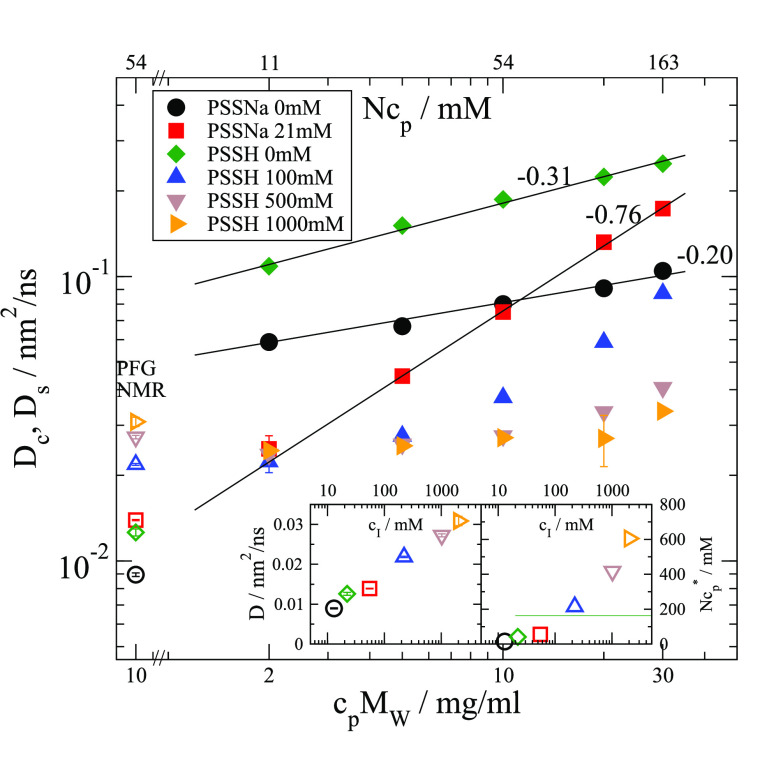
Collective diffusion coefficient *D*_c_ measured by DLS (fast mode, *Q* = 0.0264nm^–1^, solid symbols) and self-diffusion *D*_s_ (open symbols, conc. = 10 mg/ml) measured by PFG-NMR for PSS 39k. PSS type (H or Na) and added NaCl concentration *c*_s_ as given in the legend. Lines describe power laws with the indicated powers. Left inset: PFG-NMR measured self-diffusion coefficient plotted against the ion concentration *c*_I_ = 2*c*_s_ + f*Nc_p_ + 2·10^–pD^_._*c*_I_ has contributions from added salt, ions from water dissociation according to the measured pD and counterions. Because of ion condensation on the chain, an effective dissociation of f* ≈ 8% is assumed.^29^ Right inset: Monomer overlap concentration *Nc*_p_* was calculated from *D*_s_ (see SI). The line indicates the largest polymer concentration used for NSE.

For a high *c*_s_ of 500 mM and 1000 mM, we find that extrapolating *D*_c_ to dilute conditions fits well to *D*_s_, while for low *c*_s_, we observe scaling relations that cannot be used to extrapolate. For low *c*_s_, *D*_c_ are by a factor of up to 10 larger compared to *D*_s_. For the intermediate *c*_s_ 100 mM, at larger c_P_, the slope equals the scaling found for the 21 mM presenting a crossover to the respective power law regime. *D*_s_ increases with an increasing ion concentration ∼ *c*_I_^0.2^ (left inset of Figure 1). For a constant number of segments *N*, segment length *l*, and solvent viscosity η_s_, the Zimm model with *D*_*z*_ = (0.196*kT*)/(η_s_*R*_e_) implies a reduced end-to-end distance *R*_e_ = *lN*^ν^ with decreasing ν for larger *c*_I_. Electrostatic repulsion between chain segments leads to a more extended *R*_e_ for low *c*_I_. Monomer overlap concentrations *Nc*_p_* calculated from *D*_s_ are shown in the right inset of Figure 1. For *c*_I_ < 100 mM at least the larger *c*_p_ are above *c*_p_* and semidilute while others are dilute. Similar *D*_s_ increase was found for PSS 17.5k, but all concentrations were in the dilute region *c*_p_ < *c*_p_* (see SI).

Figure 2 presents small-angle X-ray scattering (SAXS) data for PSS 39k (17.5k and fit parameters, see SI). The scattered intensity is *I(Q) = c*_p_*F(Q,c*_p_*)S(Q,c*_p_*) + d(Q)* with formfactor *F(Q,c*_p_*)* and structure factor *S(Q,c*_p_*).* Because the PE dissociated ions contribute to charge screening, the formfactor depends on *c*_P_. To extract *F(Q,c*_p_*)* and *S(Q,c*_p_*)* a self-consistent simultaneous fit is used. The chain formfactor is a Gaussian chain^30^ with excluded volume parameter ν describing the chain extension complemented by a disc like cross section as we find a clear signature of a wormlike shape (see SI). At intermediate *Q*, we find a *Q*^–2^ power law, expected for Gaussian chains, followed by a clear decay described by a disc like cross section. The increase at even larger *Q* is due to diffuse scattering *d(Q)* resulting from deviations from a smooth wormlike shape. The respective disc radius of ≈0.8 nm equals the extension of PSS sidechains. Data from small angle neutron scattering (SANS) present a flatter power law (see SI). We conclude that the different contrast in SAXS and SANS is due to Na^+^ ion condensation at the chain. Adsorbed Na^+^ influences the neutron contrast less, which is dominated by hydrogens. Ion condensation also affects the effective charge of the PE chain leading to effective dissociation of ≈8%.^29^

**Figure 2:**
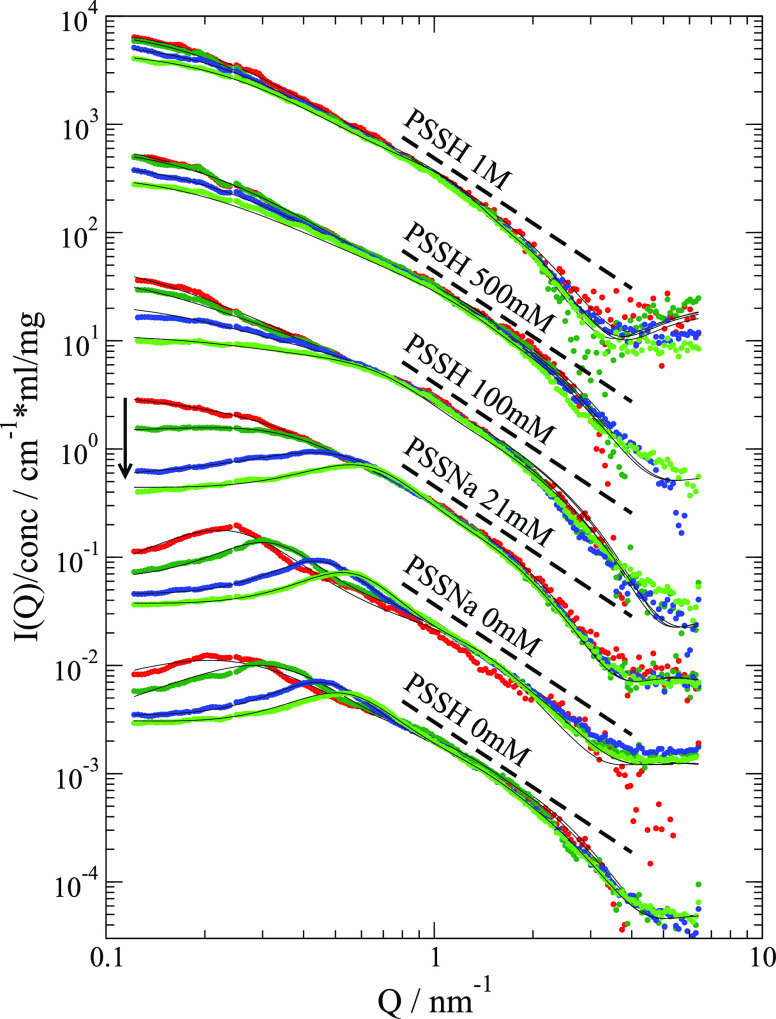
SAXS data for PSS 39k after background correction and scaled by concentration (*c*_s_ shifted consecutively by 10 for visibility). A stronger SF results mainly in the decreased intensity at a low *Q* for increased *c*_p_. Concentrations were 5, 10, 20, 30 mg/mL except for 21 mM with 8.3, 16.6, 33.2 and 50 mg/mL (colors red to green, along arrow). Black lines correspond to fits as described. For the two highest NaCl concentrations the repulsive part in 2Y SF was fixed to a negligible contribution. Broken lines indicate the extension of a *Q*^–2^ power law to a high *Q* to demonstrate the deviation from a Gaussian chain. Measured at in-house instrument SAXSpace (Anton-Paar).

*S*(*Q*) is described by a two-Yukawa (2Y) SF^31^ with screened electrostatic repulsion on a scale of a few nm and a second short-ranged attraction (<0.3 nm) (see Figure 2 and SI). We attribute the attraction to the hydrophobic backbone, to attraction mediated by Na^+^ ions condensed at the chain or excluded volume screening.^2^ We observe for increasing ion concentration, respectively, electrostatic screening a weaker SF. The 2Y SF was the only SF that reproduced the experimental PE concentration also at high ion concentration.

Figure 3 presents the dynamic structure factor of a PE chain measured by NSE^32^ in a time range from 0.1 ns to 100 ns together with a synthetic data set matching the DLS measured *D*_c_. To get an easier overview and later respective models, we determine an effective diffusion coefficient *D*_eff_ by fitting ∼ exp(−*Q*^2^*D*_eff_*t*) which is shown for samples PSS 39k in Figure 4 and for PSS 17.5k in SI. At lower *Q*, we observe the crossover from collective diffusion to chain dynamics. The relaxation at short times ∼ 1–10 ns corresponding to high *Q*, allow in detail to examine the segmental relaxation.

**Figure 3:**
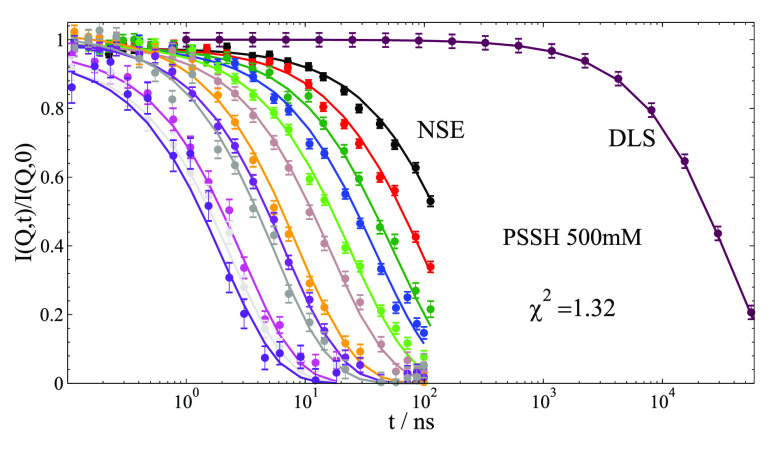
NSE measured intermediate scattering function *I*(*Q*,*t*)/*I*(*Q*,0) up to 100 ns for PSS 39k 500 mM at 30 mg/mL measured at IN15, ILL, Grenoble.^32^*Q* values are shown in Figure 4. We add a synthetic *Q* = 0.0264 nm^–1^ data set that represents the measured *D*_c_ from DLS at the same concentration (relaxation time of ≈36 μs). Solid lines represent the combined fit using the *H*(*Q*)/*S*(*Q*) corrected ZIF model with, in general, small *X*^2^. Other ion concentrations and data for PSS 17.5k are shown in SI.

**Figure 4:**
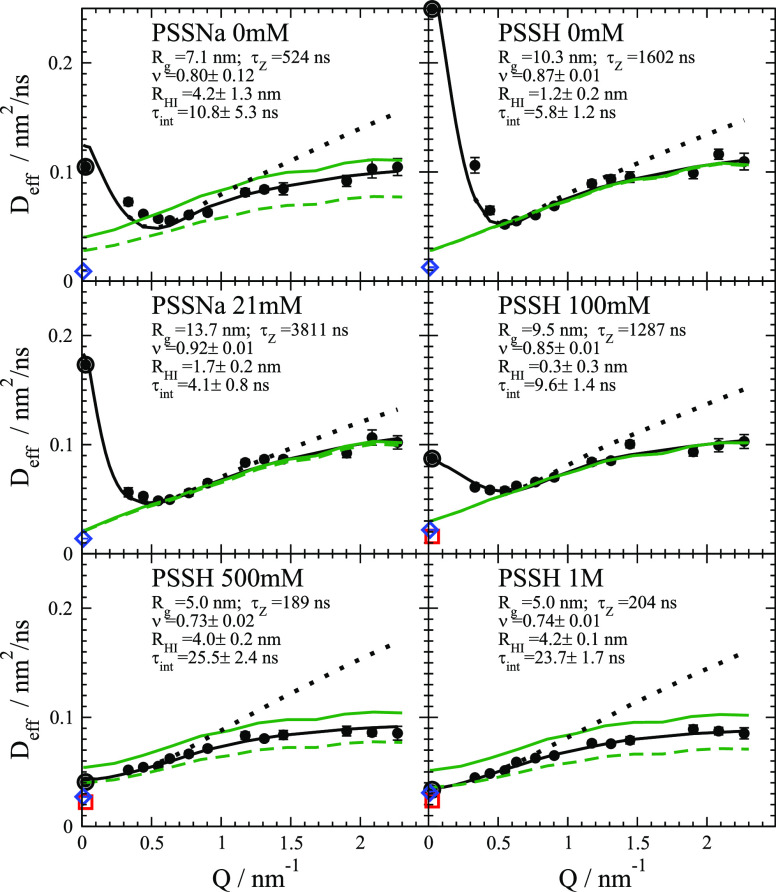
Effective diffusion coefficients *D*_eff_ for PSS 39k *c*_p_*M*_w_ = 30 mg/ml with respective parameters from the full fit model: experimental (points); ZIF with full *H*(*Q*)/*S*(*Q*) correction (black line); ZIF only with self-part correction *D*_s_/*D*_0_ (green dashed); same but τ_int_ = 0 (Zimm) (dotted); ZIF without correction (green line); DLS extrapolated concentration 0 mg/mL (red square), 30 mg/mL (circle); PFG-NMR self-diffusion (blue diamond). It should be noted that the self-part corrected ZIF (green dashed line) fits well to *D*_s_. The self-part *D*_s_/*D*_0_ correction is noticeable as a difference between solid and dashed green lines.

To describe the transition from low *Q* collective dynamics to high *Q* in detail, we use a model combining the *H*(*Q*)/*S*(*Q*) correction due to interchain interactions with a classical polymer model complemented by internal friction. We assume the decoupling of chain dynamics from interchain interactions. The Zimm model describes neutral polymers by a bead-spring model of *N* beads connected by segments of length *l* including bead friction with a solvent of viscosity η_s_ and HI between beads.^22^ The intermediate scattering function (ISF) for a single chain is
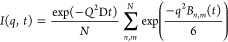
1
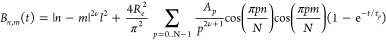
2with center of mass diffusion for a single chain *D* = *D*_*z*_ = 0.196*kT*/(η_s_*R*_e_), end-to end distance *R*_e_ = *lN*^ν^ and wavevector *Q*. The parameter *ν* describes polymer–solvent interactions and is typically between 0.5 and 0.6 for a Θ to good solvents. For charged chains, a stretching of the chain conformation is expected leading to even larger values υ > 0.8.^1,4^ The Zimm time (*p* = 1) is  and depends on the chain expansion through *R*_e_. Considering friction between neighboring beads (ξ_int_) leads to the ZIF model.^33^ Mode relaxation times  are additive slowed down by the internal friction time τ_int_ that stronger affects higher order modes observed at larger *Q*. This model was successfully used for single chain nanoparticles and IDP.^16,34^ Below the overlap concentration chain dynamics may be modified through interchain interactions.^35^

To describe the transition between *D*_c_ and intermediate *Q* NSE data, we extend the ZIF model to account for interchain interactions such as the correction for colloidal spherical particles as *D*(*Q*) = *D*_Z_*H*(*Q*)/*S*(*Q*). This implies the assumption that *S*(*Q*) and *H*(*Q*) describe a kind of configurational ensemble average and the decoupling of center of mass diffusion and intrachain dynamics. The only analytical method to calculate the hydrodynamic function *H*(*Q*) = *H*_d_(*Q*) + *D*_s_/*D*_0_ for spherical particles of radius *a* is the δγ-expansion of Beenakker and Mazur^10,11^ with the distinct contribution
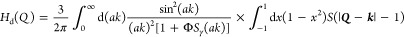
3and the self-part describing the change in self-diffusion
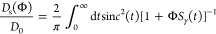
4*x* is the angle between wave vectors ***Q*** and ***k***, *S*_γ_ is a known function given by Genz and Klein.^36^ Particle correlation and the associated interactions enter the distinct part of *H*_d_ through the SF *S*(*Q*). The HI enters as the mobility of a sphere with the geometrical radius *a*. The corresponding HI volume fraction is Φ = *n*4π*a*^3^/3 with the number density *n*. For stronger interactions, e.g., charged spheres, the radius *a* and the interaction length scale are already separated. We describe the screened HI by an equivalent HI screening radius defining *R*_HI_ = *a* like attributing a hydrodynamic radius *R*_h_ to polymers or aspherical objects with an equivalent sphere diffusion coefficient. For strong HI screening, *R*_HI_ tends to be very small. For ideal Gaussian chains, *R*_h_ ≈ 0.66*R*_g_ describing the single chain friction with the solvent,^22^ which is different from *R*_HI_ that describes the effective HI between chains, but *R*_h_ might be an upper limit.

To fit the full NSE spectra, we use the SAXS measured *S*(*Q*) and calculate *H*(*Q*). The parameters for the ZIF model are fixed by the polymer dimension (*l* = 0.24 nm;^37^ 39k: *N* = 212; 17.5k *N* = 95 with *p* < 25). We fit as free parameter ν that determines *D*_Z_ and reflects chain expansion, *R*_HI_ quantifying the amount of HI screening, and τ_int_, which describes the slowing down due to internal friction. All functions are available in the used free software Jscatter.^38^

We find in general excellent fits of the full NSE spectra shown in Figure 3, and SI. Figure 4 compares *D*_eff_ of measured data, fits, and model calculations for different *c*_s_. At *Q* ≲ 0.5nm^–1^ for lower *c*_s_ the collective effect of the *H*(*Q*)/*S*(*Q*) correction is visible as an upturn, resulting in a much larger *D*_c_ compared to *D*_s_. For larger *c*_s_ the difference vanishes as *S*(*Q*) becomes weaker. Above *Q* ≈ 0.5nm^–1^ we find in general a *H*(*Q*)/*S*(*Q*) ≈ constant whereby we detect self-diffusion. We observe a Zimm like region *D* ∼ *Q* up to *Q* ≈ 1nm^–1^ with a slope close to the Zimm prediction (dotted line) for relaxation rate Γ_eff_ = *Q*^2^*D*_eff_ ∼ *Q*^3^*k*_B_*T*/η_*s*_.^22^ The linear increase in *D*_eff_ reflects *D*_Z_ and additional low mode contributions that are less influenced by internal friction. For larger *Q* deviations from Zimm dynamics due to internal friction slow down more local modes. We see for all ion concentrations that τ_int_ significantly reduces *D*_eff_ compared to that of pure Zimm. Seemingly τ_int_ increases with growing ion concentration but is still small compared to the Zimm time τ_Z_. For larger ion concentrations, the Zimm like region becomes narrower as τ_int_ increases. Inspecting Figure 3 shows that NSE is sensitive to changes on some nanosecond scale for larger *Q*. Examining *ν*, we find that with increasing ion concentration *ν* is decreasing from > 0.8 to smaller values that indicates the conformational change due to stronger screening.

PSSNa 0 mM for 39k and 17.5k shows a smaller difference between *D*_c_ and *D*_s_ compared to PSSH 0 mM or PSSNa 21 mM. This is validated by the concentration series from DLS by a reduced power law and smaller values of *D*_c_ and is not related to concentration or crossing *c*_p_*. For the 100 mM ion concentration for both, 39k and 17.5k (see SI), *R*_HI_ shows negligible values (<0.3nm) compared to significant larger values at lower and higher salt concentrations. *R*_HI_ values for 0 mM and > 100 mM are comparable. The stronger HI results in a noticeable effect from the self-part *D*_s_/*D*_0_ correction visible as difference between solid and dashed green line. Additional, *R*_HI_/*R*_g_ is close to the value *R*_h_/*R*_g_ = 0.66 expected for a Gaussian polymer, which might be a limiting case for strong HI compared to the weaker HI for intermediate ion concentrations. A weaker charge of PSSNa 0 mM compared to 21 mM can explain the reduced difference between *D*_c_ and *D*_s_ for 0 mM. This larger adsorption at lowest ion concentrations is surprising but also visible in the DLS data (see Figure 1) presenting a different power law, but it is not observed in *D*_s_. The differences can be explained by a larger counterion adsorption of Na^+^ ions on PSS at very low and high ion concentration. Na^+^ adsorption induces stronger coupling between neighboring segments, leading to larger internal friction and larger τ_Z_ for 0 mM and larger ion concentrations.

To examine the influence of temperature, we measured PSSH 0 mM, PSSNa 0 mM and PSSH 500 mM additionally at 40 and 60 °C. Resulting viscosity scaled *D*_eff_ are shown in Figure 5. The low *Q* values show a perfect scaling with solvent viscosity. For τ_int_, we see a decrease with an increasing temperature (see SI). *D*_z_ and τ_Z_ are both related to solvent viscosity, while τ_int_ is related to bond rotation, steric hindrance, charge repulsion, or Na^+^ binding between segments. The activation energy of τ_int_ is close to the water activation energy; thus, viscosity scaling also compensates for changes in τ_int_ (see SI).

**Figure 5:**
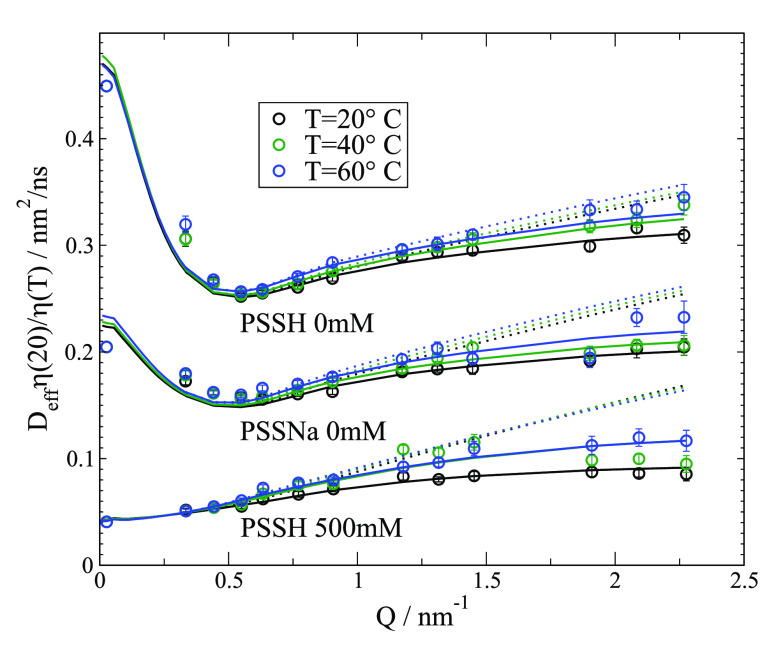
Viscosity scaled *D*_eff_ for PSS 39k at different temperatures (color coded as indicated: experimental (points); ZIF with *H*(*Q*)/*S*(*Q*) correction (solid line); same but τ_int_ = 0 (Zimm)(dotted line). All values are scaled by solvent viscosity to 20 °C. Full NSE spectra with fits and an Arrhenius plot of τ_int_ are shown in SI.

In summary, we studied the dynamics of a classical PE extending observations on macroscopic length scales to segmental chain dynamics. Different from previous hypotheses, the hydrodynamic interactions between chains are not fully screened and are essential to understand the connection between collective diffusion, self-diffusion, and chain dynamics. We observe that the dynamics of a charged PE on a segmental level follows a neutral chain Zimm model with internal friction (ZIF). The PE charges result in an increased *R*_e_. For intermediate *Q*, the chain dynamics follows the Zimm prediction, while at larger *Q*, the internal friction between neighboring monomers significantly slows segment motion. The source of internal friction seems to be related to counterion condensation.

## Supporting Information Available

DLS and PFG-NMR PSS 17.5k, Monomer overlap concentrations, SAXS model, SAXS PSS 17.5k, SAXS fit parameters, SANS data, NSE data with fits and respective fit parameters, Arrhenius behavior of internal friction (PDF)
