# Supplementary material for: Interchain Hydrodynamic Interaction and Internal Friction of Polyelectrolytes
Source: ACS Macro Lett. 2023 Aug 25;12(9):1218–23. doi: 10.1021/acsmacrolett.3c00409 (PMC10515639; doi:10.1021/acsmacrolett.3c00409)
Supplement: Supplementary file 1 — mz3c00409_si_001.pdf [file mz3c00409_si_001.pdf]

Supporting information

Interchain hydrodynamic interaction and internal friction of polyelectrolytes

Ekaterina Buvalaia,[1] Margarita Kruteva,[1] Ingo Hoffmann,[2] Aurel Radulescu,[3] Stephan Förster[1] and Ralf Biehl,[1,*]

[1]Jülich Centre for Neutron Science JCNS and Institute of Biological Information Processing IBI, Forschungszentrum Jülich GmbH, 52425 Jülich, Germany
[2]Institut Max von Laue-Paul Langevin (ILL), 71 Avenue des Martyrs, CS 20156, F-38042 Grenoble Cedex 9, France
[3]Jülich Centre for Neutron Science JCNS at Heinz Maier-Leibnitz Zentrum (MLZ), Forschungszentrum Jülich GmbH, 85748 Garching, Germany

[*] Corresponding author: ra.biehl@fz-juelich.de

Collective diffusion PSS 17.5k

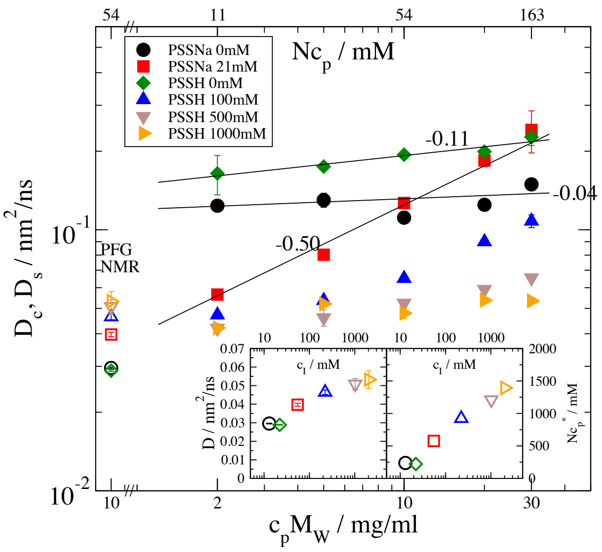

Figure S1 Collective diffusion coefficient D_c measured by DLS (fast mode, solid symbols) and self-diffusion D_s (open symbols, conc.=10 mg/ml) measured by PFG-NMR for PSS 17.5k. The PFG-NMR X-axis position reflects a low concentration where D_c should equal D_s. Lines describe power laws with indicated powers. The inset A shows the PFG-NMR measured self-diffusion D_s plotted against the ion concentration assuming a PSS effective dissociation rate of 8%[1] and ions from water dissociation according to a measured pD 1.8 at 10mg/ml. Right inset: Monomer overlap concentration Nc_p^* calculated from D_s (see below). All concentrations are below Nc_p^*.

Overlap concentrations :

Neutral polymer solutions are categorized in 3 different concentration regimes: dilute, semidilute and entangled/concentrated[2]. In the dilute region polymers are separate and polymer interactions have a small effect. Polyelectrolytes in dilute conditions at low ionic strength show electrostatic repulsion leading to an electrostatically correlated dilute regime not present for neutral polymers. The semidilute regime is characterized by overlapping coils without entanglements of the polymer.

The crossover from dilute to semidilute happens when the occupied polymer volumes fills the sample volume. The polymer coils start to overlap at a monomer concentration $c^* \approx \frac{N}{R_g^3}$ for polymers with polymerization degree N.[3] Corresponding polymer concentration are $c_p^* = c^*/N \approx \frac{1}{R_g^3}$. Monomer overlap concentrations Nc_p are given for easier comparison to other work e.g. to Lopez[3].

From measured self-diffusion coefficient D_s we can calculate the radius of gyration R_g using $R_g^2 = 6R_e^2$ with

$$D_s = D_z = 0.196\mathrm{k}T/(\eta_s R_e)$$

and determine c_p^*

Table 1: Monomer overlap concentrations Nc_p^* from D_s measured at 10m/ml in units mM. For 30mg/ml the additional counterions and changed pD are considered by linear interpolation of 10 mg/ml data. The monomer concentration for later NSE measurements 39k and 17.5k is 163 mM corresponding to 30mg/ml.

sample	PSSNa 0 mM	PSSNa 21 mM	PSSH 0mM	PSSH 100 mM	PSSH 500 mM	PSSH 1M
c_I at 10mg/ml mM	13	55	22	222	1022	2022
Nc_p^* for 39k	14	55	41	214	416	605
Nc_p^* for 17.5k	238	577	221	922	1209	1392
c_I at 30mg/ml mM	41	83	50	250	1050	2050
Nc_p^* for 39k	49	82	53	222	421	-
Nc_p^* for 17.5k	440	636	534	933	1214	-

Lopez examined scattering data (SAXS and LS) and viscosity data of PSSNa for different polymerization degree N yielding master curves of $c^* = 1750N^{-2}$ and $c^* = 3600N^{-2}$ at low ionic strength[3]. For N = 212 these results in Nc_p^* values of 39 mM or 80 mM while for N=95 resulting values are 194 mM and 400 mM, respectively. We find reasonable agreement to these values considering that lowest ionic strength is difficult to achieve.

SAXS model

Generalized Gaussian worm.

The formfactor of a generalized Gaussian chain is[4]

$$F_g(Q, R_g, \upsilon) = \frac{1}{\upsilon U^{\frac{1}{2\upsilon}}}\gamma_{inc}\left(\frac{1}{2\upsilon}, U\right) - \frac{1}{\upsilon U^{\frac{1}{\upsilon}}}\gamma_{inc}(\frac{1}{\upsilon}, U)$$

with $U = (QR_g)^2$ and the lower incomplete gamma unction γ_{inc}. R_g is the radius of gyration and υ the excluded volume parameter.

If the contour length L is much larger than the cross section, the cross section can be separated in a decoupling approximation like the for a wormlike chain[5]. The Gaussian worm formfactor reads:

$$F(Q) = \rho^2 V^2 F_g(Q, R_g, \upsilon) \cdot F_{disc}(Q, R)$$

with $F_{disc}(Q,R) = \frac{J_1(QR)}{QR}$, $J_1(x)$ as first order Bessel function and ρ as contrast of the worm with volume $V = \pi R^2 L$.

Two Yukawa SF

The double Yukawa potential of particles with radius R at distance r' is

$$V(r)/kT = \begin{bmatrix} \infty & r \leq 1 \\ -K_1\left[\frac{e^{-z_1(r-1)}}{r}\right] - K_2\left[\frac{e^{-z_2(r-1)}}{r}\right] & r > 1 \end{bmatrix}$$

with $z_i=1/\sigma_I$ as inverse screening length (σ_I as screening length), reduced distance r=r'/R and potential K_i at the surface. For K>0 we have attraction while K<0 means repulsion.

Liu et all describe the corresponding structure factor S(Q) within the MSA closure.[6]

Diffuse scattering

Diffuse scattering d(Q) describes contributions from inhomogeneities or deviations from a smooth shape like the Gaussian worm assuming statistical independence and a characteristic length σ. [7]

$$d(Q,\sigma) = A(1 - e^{-Q^2\sigma^2})$$

SAXS 17.5k

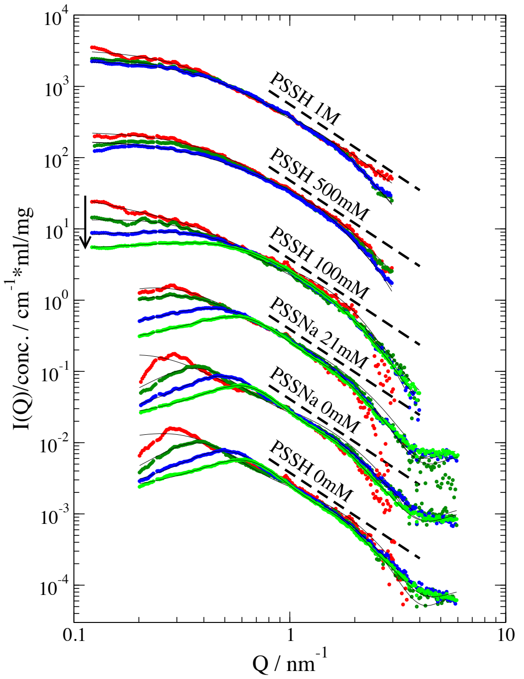

Figure S2 SAXS data for PSS 17.5k. All measurements were background corrected and scaled by concentration (c_s shifted consecutively by 10 for visibility).). Concentrations were 5, 10, 20, 30mg/ml (colors red to green, along arrow) Black lines correspond to fits as described in main text. See Figure 2 in main text.

SAXS fit parameters

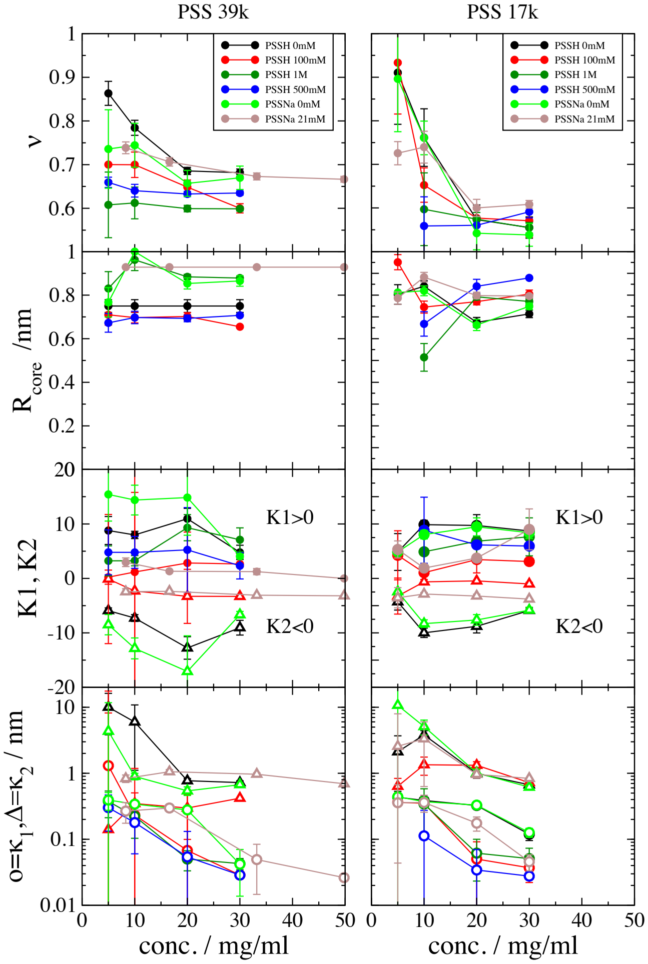

Figure S3 Relevant SAXS fit parameters for the generalized Gaussian worm (R_{core}, excluded volume parameter υ) and for the two-Yukawa potential (screening length κ_I, surface potential K_i in units kT). $K_1>0$ with short range κ_1 describes attraction, while K_2 describes electrostatic repulsion. Characteristic length of diffuse scattering σ resulted in values between 0.2-0.9 nm of varying intensity which might also include background contributions,

SANS

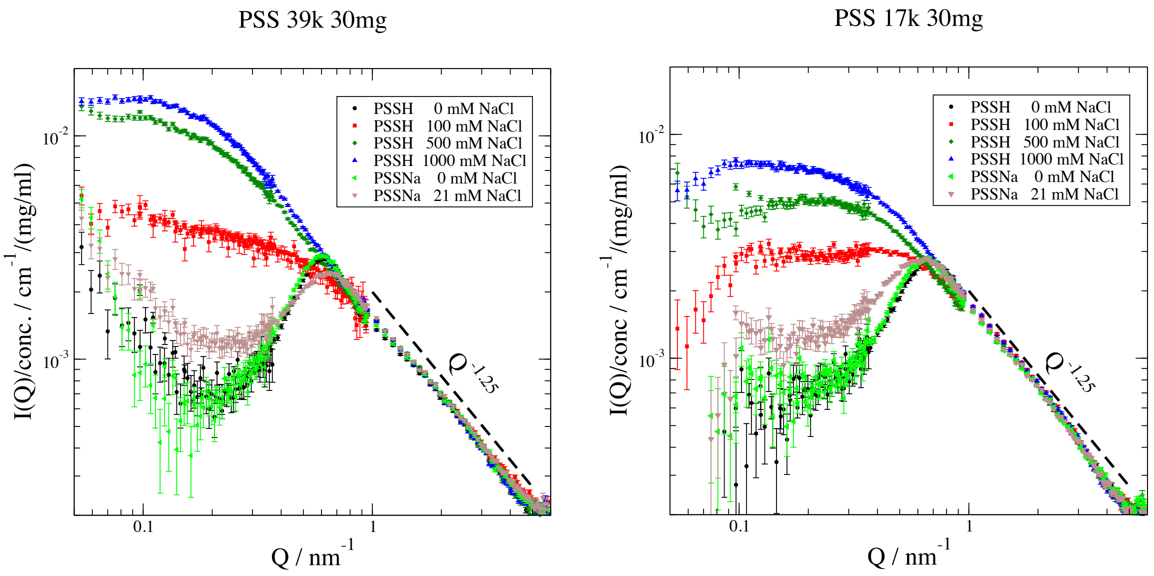

Figure S4: SANS data at T=20ºC for PSS 39k (left) and 17.5k (right) after background correction and desmearing measured at KWS2, MLZ, Garching[8]. For 17.5k a small increase in slope at larger Q is visible that might also be an indication of the disclike formfactor observed in SAXS. For 39k at low Q we observe for low salt concentrations an upturn caused by the presence of aggregates also visible in DLS as the slow mode. Extrapolating this contribution to the NSE Q range shows that the slow mode is negligible in the NSE signal. For 17.5k the contribution is even smaller. The data are corrected for instrumental resolution using the Lake algorithm that instrumental resolution does not affect the shown data or the observed power law.

NSE

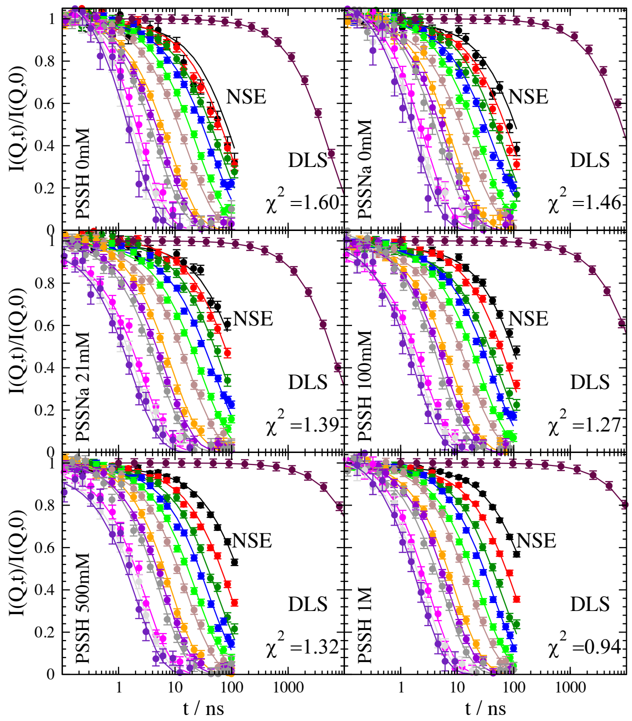

Figure S5 NSE measured intermediate scattering function I(Q,t)/I(Q,0) up to 100 ns for PSS 39 kDa at 30 mg/ml measured at IN15, ILL, Grenoble. Q values as shown in ***Error! Reference source not found.*** *(main text). We add a synthetic Q=0.0264 nm^{-1} dataset that represents a field correlation function $g_1(Q,t)$ with the measured collective diffusion coefficient from DLS at the same concentration. The synthetic dataset was created with same number of points as the lowest Q NSE measurement and approximately same error bars. This results in a weighing of the DLS values equal to the lowest NSE Q value together with a small influence on X^2. Solid lines represent the combined fit using the H(Q)/S(Q) corrected ZIF model with in general small X^2.*

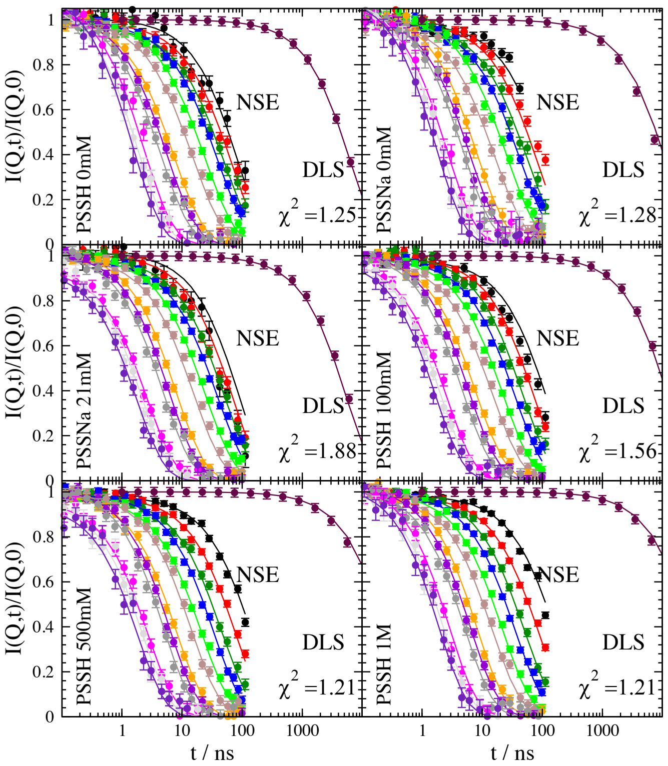

Figure S6 NSE measured intermediate scattering function I(Q,t)/I(Q,0) up to 100 ns for PSS 17.5 kDa at 30 mg/ml measured at IN15, ILL, Grenoble. See description Figure S5.

Effective diffusion

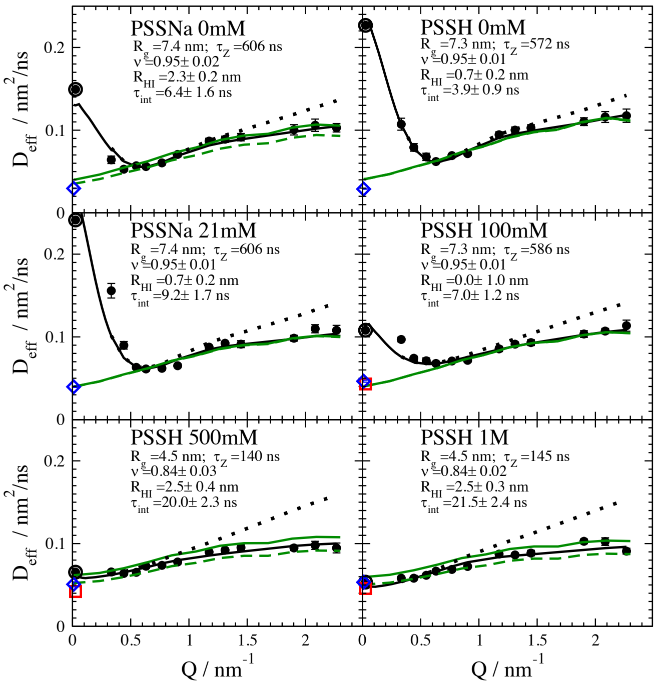

Figure S7 Effective diffusion coefficients D_{eff} for PSS 39kDa with respective parameters from the full fit model: experimental (points); ZIF with full H(Q)/S(Q) correction (black line); ZIF only with self-part correction D_s/D_0 (green dashed); same but τ_{int}=0 (ZIMM) (dotted); ZIF without correction(green line); DLS extrapolated concentration 0mg/ml (red square), 30m/ml (circle);;PFG-NMR self-diffusion (blue diamond).

Temperature effect

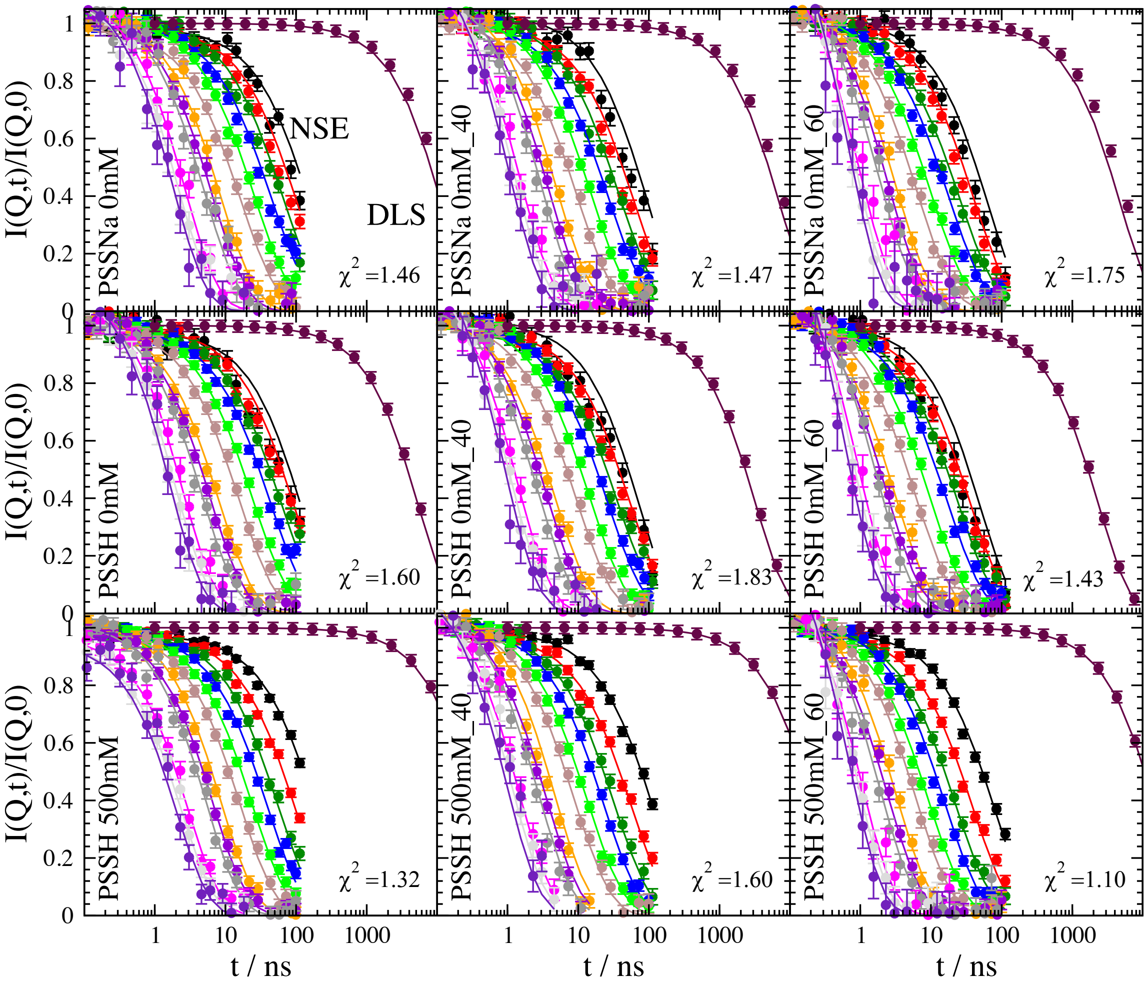

Figure S8: NSE measured intermediate scattering function I(Q,t)/I(Q,0) up to 100 ns for PSS 39 kDa at 30 mg/ml measured at IN15, ILL, Grenoble for 20, 40and 60 ºC (from left to right) . We add a synthetic Q=0.0264 nm^{-1} *dataset that represents the measured collective diffusion coefficient from DLS at the same. Solid lines represent the combined fit using the H(Q)/S(Q) corrected ZIF model with in general small* X^2.

Arrhenius behavior of internal friction

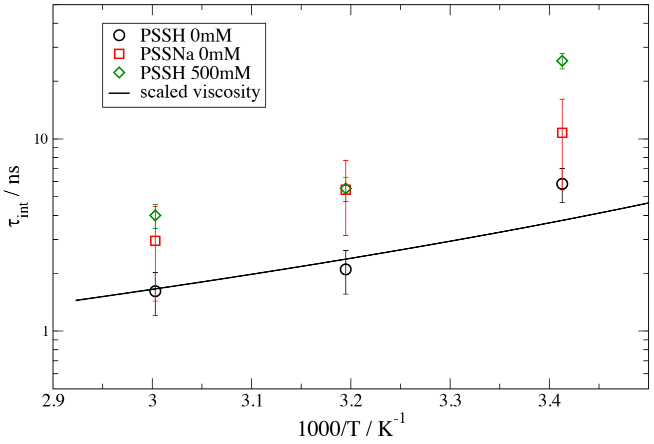

Figure S9: Arrhenius behavior of internal friction in comparison to D_2O viscosity. Viscosity is scaled by an arbitrary factor 3000. The activation energy of water viscosity is 15.7 kJ/mol[9]. Similar slopes indicate a similar activation energy.

REFERENCES

(1) Jacobs, M.; Lopez, C. G.; Dobrynin, A. V. Quantifying the Effect of Multivalent Ions in Polyelectrolyte Solutions. *Macromolecules* **2021**, *54* (20), 9577–9586. https://doi.org/10.1021/acs.macromol.1c01326.

(2) Doi, M.; Edwards, S. F. *The Theory of Polymer Dynamics*; Birman, J., Edwards, S. F., LLewellyn Smith, C. H., Rees, M., Eds.; Oxford University Press, USA: Oxford, 1988. https://doi.org/10.1016/S1359-0286(96)80106-9.

(3) Lopez, C. G. Scaling and Entanglement Properties of Neutral and Sulfonated Polystyrene. *Macromolecules* **2019**, *52* (23), 9409–9415. https://doi.org/10.1021/ACS.MACROMOL.9B01583/ASSET/IMAGES/LARGE/MA9B01583_0001.JPEG.

(4) Hammouda, B. Analysis of the Beaucage Model. *J Appl Crystallogr* **2010**, *43* (6), 1474–1478. https://doi.org/10.1107/S0021889810033856.

(5) Jerke, G.; Pedersen, J. S.; Egelhaaf, S. U.; Schurtenberger, P. Static Structure Factor of Polymerlike Micelles: Overall Dimension, Flexibility, and Local Properties of Lecithin Reverse Micelles in Deuterated Isooctane. *Phys Rev E* **1997**, *56* (5), 5772. https://doi.org/10.1103/PhysRevE.56.5772.

(6) Liu, Y.; Chen, W. R.; Chen, S. H. Cluster Formation in Two-Yukawa Fluids. *Journal of Chemical Physics* **2005**, *122* (4), 044507. https://doi.org/10.1063/1.1830433.

(7) Förster, S.; Timmann, A.; Konrad, M.; Schellbach, C.; Meyer, A.; Funari, S. S.; Mulvaney, P.; Knott, R. Scattering Curves of Ordered Mesoscopic Materials. *J Phys Chem B* **2005**, *109* (4), 1347–1360. https://doi.org/10.1021/jp0467494.

(8) Radulescu, A.; Szekely, N. K.; Appavou, M.-S. KWS-2: Small Angle Scattering Diffractometer. *Journal of large-scale research facilities JLSRF* **2015**, *1*, A29. https://doi.org/10.17815/jlsrf-1-27.

(9) Messaâdi, A.; Dhouibi, N.; Hamda, H.; Belgacem, F. B. M.; Adbelkader, Y. H.; Ouerfelli, N.; Hamzaoui, A. H. A New Equation Relating the Viscosity Arrhenius Temperature and the Activation Energy for Some Newtonian Classical Solvents. *J Chem* **2015**, *2015*, 163262. https://doi.org/10.1155/2015/163262.
